# Supplementary material for: Long-Term Prognosis after Coronary Artery Bypass Grafting: The Impact of Arterial Stiffness and Multifocal Atherosclerosis
Source: J Clin Med. 2022 Aug 5;11(15):4585. doi: 10.3390/jcm11154585 (PMC9369624; doi:10.3390/jcm11154585)
Supplement: Supplementary file 1 [file jcm-11-04585-s001.zip › jcm-1821447-supplementary.pdf]

Suppl. Table S1. – Cause of death in both groups in the long-term period after CABG

Variables	Group 1 CAVI<9,0 (n=163)		Group 2 CAVI≥9,0 (n=111)		p
	n	%	n	%	
**Death**	**37**	**22.7**	**39**	**35.14**	**0.023**
**Cardiovascular:**	**19**	**11.6**	**25**	**22.52**	**0.016**
Myocardial infarction	8	5.0	7	6.3	0.61
Stroke	7	4.2	2	1.8	0.25
Pulmonary embolism	0	0	1	0.9	0.2
Other cardiovascular	5	3.1	14	12.7	0.004
**Non-cardiovascular:**	**13**	**7.9**	**12**	**10.8**	**0.42**
Oncology	8	4.9	6	5.4	0.83
Covid	2	1.22	2	1.8	0.69
Trauma	0	0	1	0.9	0.2
Other	3	1.82	1	1.02	0.9
**Unknown**	**5**	**3.07**	**2**	**1.8**	**0.51**
